# Supplementary material for: Multi-population genetic algorithm with ER network for solving flexible job shop scheduling problems
Source: PLoS One. 2020 May 29;15(5):e0233759. doi: 10.1371/journal.pone.0233759 (PMC7259647; doi:10.1371/journal.pone.0233759)
Supplement: Attachment — Submitted filename: Comments.docx [file pone.0233759.s002.docx]

**Comments**

I want to thank the author for submitting the paper “Multi-population genetic algorithm with ER network to solve flexible job shop scheduling problem”. Even though I find the research idea and the topic in general quite interesting and relevant, I have many concerns that should be addressed.:

- Most of this work is already published in previous work (Shi, Long et al. 2020). As an extension, the authors solved more FJSP instances and compared the obtained results with previous works for the same instances. The authors should clearly explain the similarity and differences between this research and the one mentioned above.
- The methodology of the study should be clearly elaborated, in order to argue your decisions and to remove the doubts.
- In lines 15-25, the authors stated that they study the effects of sub-population size and number and the propagation rate of advantageous genes on the performance of MPGA-ER. However, they did not define what the performance is.
- The language structure needs to be improved.
- Sections are not numbered.
- In lines 17 -18, the author mentioned that “the performance shows considerable improvement compared to the traditional GA”. However, this comparison is not found in the work.
- The study mentioned ER network as the main contribution, however, it is not defined clearly.
- In line 277, it was mentioned: “The average of the obtained optimal values termed as AOV is used to evaluate the performance of MPGA-ER”. The authors did not clearly define the optimal values.
- In line 280 an equation of success rate was introduced “eq. 7”, “where Nsuc denotes the number of times MPGA-ER can find the optimal value of the corresponding FJSP problem among Ntol independent runs” how the authors guarantee that the solution is optimal?
- What is the “average optimal value”?
- In line 425, it was mentioned that “According to Sections 3.1~3.4”. These numbers were not presented.
- In line 426, the reported optimization parameters of MPGA-ER are Pr = 0.08, P = 0.009, S = 100, N = 80, R = 3, and IT = 1000, however various GA parameters have not optimized.
- In Table 4, the authors compared the results with previous works only in terms of “optimal values” (Cmax). However, the CPU times were not presented.
- In Table 4, the authors compared the results with previous works in terms of “optimal values”. Referring to one of these works, [31], these results are the best-obtained solutions (but not proofed to be optimal) as shown below

- In the conclusion section (line 506), it was mentioned: “Unlike previous studies, to obtain an improved MPGA-ER, network connectivity is not necessary”. However, this conclusion was not clearly explained in the results section.

I hope the authors will find my comments constructive and helpful.

Shi, X., W. Long, Y. Li, D. Deng and Y. Wei (2020). "Research on the performance of multi-population genetic algorithms with different complex network structures." Soft Computing: 1-19.
