# Supplementary material for: Multi-population genetic algorithm with ER network for solving flexible job shop scheduling problems
Source: PLoS One. 2020 May 29;15(5):e0233759. doi: 10.1371/journal.pone.0233759 (PMC7259647; doi:10.1371/journal.pone.0233759)
Supplement: Attachment — Submitted filename: Response to Reviewers.docx [file pone.0233759.s003.docx]

Replies to the comments

Dear Editors and Reviewers,

We would like to express our sincere gratitude for all of you.

Thank you for your letter and for the reviewers’ comments concerning our manuscript entitled “Multi-population genetic algorithm with ER network for solving flexible job shop scheduling problems.” We have studied comments carefully and made some changes. The main changes in the manuscript and the responds to the reviewers’ comments are as flows.

**Reviewer #1**

In the study “Multi-population genetic algorithm with ER network to solve flexible job shop scheduling problem”, the authors propose a multi-population GA with ER network to solve the flexible job shop scheduling problem. They can show that their developed algorithm can dominate the traditional GA.

This topic is interesting. However, a major revision is needed to improve the paper quality. Some important comments are given below:

1. The writing and English should be improved. Pay careful attention to the usage of articles. Moreover, you can’t use “research” as a countable noun.

**Responses**: According to your comments, we have improved our writing and English language carefully by us. We also ask “Editage” for a professional English language editing.

2. Number the sections and subsections for better positioning.

**Responses**: According to your comments, we have numbered the sections and subsections.

3. This topic has a widespread background. So, I suggest the authors use a tabular form for better comparisons.

**Responses**: According to your comments, we have used a Table to compare several evolutionary algorithms with different networks. (see Table 1 in the manuscript)

4. Add the domain of variables to the mathematical model of FJSP.

**Responses**: According to your comments, we have added the domains of all variables to our mathematical model. (see Equations (1)~(10))

5. My major concern is regarding the novelty of the algorithm, which combines a number of methods. Two questions are raised to me. 1) Why these methods are selected rather than others? 2) What are the specialties for the studied problem?

**Responses**: To avoid premature convergence which is the main disadvantage of the standard genetic algorithm (GA), we used the multi-population method and ER network to improve GA, obtaining the multi-population GA with ER network (MPGA-ER) in this study. We think there are several reasons why we choose GA that is improved by multi-population method: 1) the GA is simplicity, easy realization, and wide applicability; 2) in our previous research [4], we have found that the performance of GA can be improved by using multi-population method solely; and 3) we are familiar with genetic algorithm. There are some reasons why we choose ER network to design multi-population GA (MPGA) as follows. In our previous research [17], we have used seven networks including ER network to design MPGA to address how different networks affect the performance of MPGA, where the connection probability (*P*) of the ER model was a constant. We found that the propagation rate of advantageous genes among sub-populations was limited by these network topologies; hence, how the propagation rate over a wide range affects the performance of MPGA is still not clear. Fortunately, when the connection probability of the ER model changes from 0 to 1, the corresponding network gradually transforms from a graph that completely includes isolated points to a complete graph; therefore, the propagation rate of the advantageous genes changes from considerably low to high. Accordingly, to know how the propagation rate over a wide range affects the performance of MPGA, we used the multi-population method and ER network to improve GA. We think this may be the specialties of our study. According to your comments, these reasons are described in our revised manuscript directly or indirectly. (see the words in red color in Section 1 of the version named “Revised Manuscript with Track Changes”)

6. How could you set the parameters of the algorithm?

**Responses**: We set the parameters of the algorithm according to our simulations.

7. You should explain the main limitations of your proposed algorithm. Moreover, the practical aspect of the problem you studied should be discussed in more detail.

**Responses**: According to your comments, we have explained the main limitations of our algorithm in the Conclusion Section. We also discussed some practical applications of our study in the Conclusion Section.

8. The authors may read and consider the following references to improve the introduction of the manuscript.

- https://doi.org/10.1016/j.cie.2019.106007

- https://doi.org/10.1080/21693277.2019.1620651

- https://doi.org/10.3390/su10051366

**Responses**: According to your comments, we have read all the three papers you give us carefully to improve our introduction. (see the words in red color in Sections 1 and 2 of the version named “Revised Manuscript with Track Changes”)

Finally, thank you very much, and your comments are constructive and helpful.

**Reviewer #2**

I want to thank the author for submitting the paper “Multi-population genetic algorithm with ER network to solve flexible job shop scheduling problem”. Even though I find the research idea and the topic in general quite interesting and relevant, I have many concerns that should be addressed:

1. Most of this work is already published in previous work (Shi, Long et al. 2020). As an extension, the authors solved more FJSP instances and compared the obtained results with previous works for the same instances. The authors should clearly explain the similarity and differences between this research and the one mentioned above.

**Responses**: In our previous research [17] that you mentioned, we have used seven different networks including ER network to design MPGA (multi-population genetic algorithm), thereby addressing how different networks affect the performance of MPGA. However, in [17], the connection probability (*P*) of the ER model was a constant. We found that the propagation rate of advantageous genes among sub-populations was limited by these network topologies; hence, how the propagation rate over a wide range affects the performance of MPGA is still not clear. Fortunately, when the connection probability of the ER model changes from 0 to 1, the corresponding network gradually transforms from a graph that completely includes isolated points to a complete graph; therefore, the propagation rate of the advantageous genes changes from considerably low to high. Accordingly, as an extension of [17], to know how the propagation rate over a wide range affects the performance of MPGA, we used the ER network to improve GA. These reasons are added to our revised manuscript directly or indirectly. (see the words in red color in Section 1 of the version named “Revised Manuscript with Track Changes”)

2. The methodology of the study should be clearly elaborated, in order to argue your decisions and to remove the doubts.

**Responses**: According to your comments, the methodology of this study has been read and the revised carefully, such as the description of ER model. (see the words in red color in Section 2 of the version named “Revised Manuscript with Track Changes”)

3. In lines 15-25, the authors stated that they study the effects of sub-population size and number and the propagation rate of advantageous genes on the performance of MPGA-ER. However, they did not define what the performance is.

**Responses**: According to your comments, we have defined what the performance is in the Abstract Section. (see the words in red color in Abstract Section of the version named “Revised Manuscript with Track Changes”)

4. The language structure needs to be improved.

**Responses**: According to your comments, we have improved our English language carefully by us. We also ask “Editage” for a professional English language editing.

5. Sections are not numbered.

**Responses**: According to your comments, we have numbered the Sections and Sub-sections in our revised manuscript.

6. In lines 17 -18, the author mentioned that “the performance shows considerable improvement compared to the traditional GA”. However, this comparison is not found in the work.

**Responses**: This results is implied as follows: as shown in Table 3, the performance of MPGA-ER when *P* = 0 (equivalent to 100 standard GAs running simultaneously) is not better than that of MPGA-ER when *P* = 1, let alone that of MPGA-ER when *P* = 0.009 which is the best, implying that the performance shows considerable improvement compared to the traditional GA. These descriptions have been added in our revised manuscript. (see the words in red color in Section 4 of the version named “Revised Manuscript with Track Changes”)

7. The study mentioned ER network as the main contribution, however, it is not defined clearly.

**Responses**: According to your comments, we have defined the ER model more clearly. (see the words in red color in Section 2.2 of the version named “Revised Manuscript with Track Changes”)

8. In line 277, it was mentioned: “The average of the obtained optimal values termed as AOV is used to evaluate the performance of MPGA-ER”. The authors did not clearly define the optimal values.

**Responses**: Actually, there are two concepts of optimal values in our study, which are not defined clearly. We feel sorry about it. The optimal value in the concept of AOV refers to the best solution of the corresponding FJSP problem found by the algorithm in a single run. In addition, we have added an equation to define AOV (Equation (13)). On the other hand, the optimal value in the concept of SR refers to the best solution of the corresponding FJSP problem can be found so far in the literature. And these descriptions are added to our revised manuscript. (see the words in red color in Section 2.4 of the version named “Revised Manuscript with Track Changes”)

9. In line 280 an equation of success rate was introduced “eq. 7”, “where Nsuc denotes the number of times MPGA-ER can find the optimal value of the corresponding FJSP problem among Ntol independent runs” how the authors guarantee that the solution is optimal?

**Responses**: Here, the optimal value refers to the best solution of the corresponding FJSP problem can be found so far in the literature. Actually, the optimal value means the best solution of the corresponding FJSP problem can be found so far in the literature, which we defined it as optimal value.

10. What is the “average optimal value”?

**Responses**: According to your comments, we have added an equation to define AOV (Equation (13)).

11. In line 425, it was mentioned that “According to Sections 3.1~3.4”. These numbers were not presented.

**Responses**: According to your comments, we have numbered all the Sections.

12. In line 426, the reported optimization parameters of MPGA-ER are Pr = 0.08, P = 0.009, S = 100, N = 80, R = 3, and IT = 1000, however various GA parameters have not optimized.

**Responses**: The parameters *Pr* and *IT* are indeed ignored in our study. The parameter *Pr* of GA is used to increase population diversity, but cannot be set too large. In this study, we mainly use multi-population to increase the diversity of GA. Thus, the parameter *Pr* is set as a small value randomly in this study. When considering TIN, the parameter *IT* can be calculated approximately when other parameters are given. In our previous research [4], we addressed how different TINs (associated with IT) affect the performance of MPGA. We found that it cannot affect the performance of MPGA when it is large (larger than 450,000 according to [4]). When it is very small, with increase of TIN, the performance of MPGA becomes better. Thus, the parameter *IT* is set as a large number randomly in this study.

13. In Table 4, the authors compared the results with previous works only in terms of “optimal values” (Cmax). However, the CPU times were not presented.

**Responses**: For fairly, we did not realize these algorithms in other papers by us and cited the results directly from the original papers. Thus, the CPU times may become meaningless and were not given.

14. In Table 4, the authors compared the results with previous works in terms of “optimal values”. Referring to one of these works, [31], these results are the best-obtained solutions (but not proofed to be optimal) as shown below.

**Responses**: The optimal value here also refers to the best solution of the corresponding FJSP problem can be found so far in the literature.

15. In the conclusion section (line 506), it was mentioned: “Unlike previous studies, to obtain an improved MPGA-ER, network connectivity is not necessary”. However, this conclusion was not clearly explained in the results section.

**Responses**: According to your comments, we have explained this conclusion carefully in the Section 4. (see the words in red color in Section 4 of the version named “Revised Manuscript with Track Changes”)

Finally, thank you very much, and your comments are constructive and helpful.

Thank you and beat regards.

Yours sincerely

Xiaoqiu Shi; Wei Long; Yanyan Li; Dingshan Deng
