# Supplementary material for: Multi-population genetic algorithm with ER network for solving flexible job shop scheduling problems
Source: PLoS One. 2020 May 29;15(5):e0233759. doi: 10.1371/journal.pone.0233759 (PMC7259647; doi:10.1371/journal.pone.0233759)
Supplement: Attachment — Submitted filename: Comments_R2.docx [file pone.0233759.s004.docx]

The authors addresses all the previous comments, however, still there is a need for explaination, and there are some other comments.

The manuscript was written carelessly. Some examples:

- In abstract: “Finally, we use a parameter-optimized MPGA-ER to solve for more FJSP instances and demonstrate its effectiveness by comparing it with other algorithms.” Did the authors propose different algorithms? Besides, In Table 5, there are no algorithms presented in columns 4-6 or even in the discussion.
- No consistency in writing for example in line 249 “(i) Machine crossover: two integers less than” and in line 251 “(ii)Operation crossover is POX crossover”. The same also in line 264 ‘(i) Machine mutation:” and no number in line 268 “Operation mutation:”
- No consistency in writing for example in line 313 “Finally, the measures: average shortest path (ASP) and size of the maximum connected sub-graph (SMCS) are used to measure the ER network structures.”. However, the authors started describing SMCS first.
- In section 2.3, point (3) mutation: different crossover operators were presented and the caption of Figure 2 is Fig 2. Schematic diagram of a crossover. Which one of them is that?
- In abstract: ”we study how the sub-population size and number and the propagation rate of advantageous genes affect the performance (evaluated by the average optimal value and success rate based on TIN) of MPGA-ER. Is it appropriate to insert the sentence “(evaluated by the average …) in this way?

In line 144, there is no reference for the mathematical model.

Symbols of FJSP should be tabulated.

The authors did not show a Gantt chart of an arbitrary schedule to the example presented in Table 2.

What is J_t_ in Line 214?

What is POX crossover stands for?

The two-point crossover was not explained and a figure is needed to present it.

There is a reference ([27]) in line 251 (“Operation crossover is POX crossover [27]”). This refers to what.

Subsections numbers of 2.3 section.

In line 307 it was mentioned ” SR, which is also used to evaluate the performance of MPGA-ER”. However, this is the same as that in line 302.

In line 303: “For a given TIN, every algorithm runs several times (denoted by Ntol) independently”. However, this number was not stated.

A short description of how the instances were generated is preferred.
