# Supplementary material for: Multi-population genetic algorithm with ER network for solving flexible job shop scheduling problems
Source: PLoS One. 2020 May 29;15(5):e0233759. doi: 10.1371/journal.pone.0233759 (PMC7259647; doi:10.1371/journal.pone.0233759)
Supplement: Attachment — Submitted filename: Response to Reviewers.docx [file pone.0233759.s005.docx]

Replies to the comments

Dear Editors and Reviewers,

We would like to express our sincere gratitude for all of you.

Thank you for your letter and for the reviewers’ comments concerning our manuscript entitled “Multi-population genetic algorithm with ER network for solving flexible job shop scheduling problems.” We have studied comments carefully and made some changes. The main changes in the manuscript and the responds to the reviewers’ comments are as flows.

**Reviewer #1**

Good work. The authors tried to improve the quality of the manuscript based on my previous comments. However, there are still two minor concerns to be resolved.

1. I asked the authors "How could you set the parameters of the algorithm?" I mean what was the motivation? How can you ensure these values are logical?

**Responses**: We are very sorry to misunderstand you last time. And we are trying to answer your question correctly here. In our study, all parameters of the algorithm are as follows: TIN (total individual number), *P_r_* (mutation probability), *P* (connection probability of the ER model), *S* (sub-population size), *N* (sub-population number), *R* (a parameter used to control the migration frequency), and IT (iteration times). How the parameters *P*, *S*, *N*, and *R* affect the performance of the algorithm is our theme in this study and hence they are studied by using simulation. And we think these parameters are set reasonably. The parameters *P_r_*, TIN and IT are indeed ignored in our study. The parameter *P_r_* of GA is used to increase population diversity, but cannot be set too large. In our study, we mainly use multi-population to increase the diversity of GA. Thus, the parameter *P_r_* is set as a small value (0.08) randomly in our study. The parameter TIN was studied in our previous research [4]. We found that it cannot affect the performance of MPGA when it is very large (larger than 450,000 according to [4] for the same FJSP instance). When it is very small, with increase of TIN, the performance of MPGA becomes better. Thus, it is ignored and set to be a large number. In [17], in which they solved the same FJSP instance, the TIN was 1,600,000. To provide more chances for communication among sub-populations, we set TIN to be 2,000,000 in this study randomly. The parameter IT (associated with TIN) is also ignored. And when it is very large it cannot affect the performance of MPGA-ER. Thus, it is also ignored and set to be a large number (1,000) randomly in Section 3.5. According to your comments, these reasons are added to the revised manuscript directly or indirectly.

2. Develop the managerial insights section and provide more useful suggestion based on the main achievements of the study.

**Responses**: According to your comments, we have tried to improve Section 4 whose title is changed from “Analysis of results” to “Analysis of results and discussion”, giving more discussion and useful suggestion in this section. (see the words in red color in Section 4 of the version named “Revised Manuscript with Track Changes”)

Finally, thank you very much, and your comments are constructive and helpful.

**Reviewer #2**

The authors address all the previous comments, however, still there is a need for explaination, and there are some other comments.

1. In abstract: “Finally, we use a parameter-optimized MPGA-ER to solve for more FJSP instances and demonstrate its effectiveness by comparing it with other algorithms.” Did the authors propose different algorithms? Besides, In Table 5, there are no algorithms presented in columns 4-6 or even in the discussion.

**Responses**: We did not propose different algorithms. The other algorithms used to compare were proposed in other papers. These algorithms were not described directly in our manuscript last time. According to your comments, these algorithms are all described directly (in both Table 6 and the discussion) in the revised manuscript. And the sentence “Finally, we use a parameter-optimized MPGA-ER to solve for more FJSP instances and demonstrate its effectiveness by comparing it with other algorithms.” is changed to “Finally, we use a parameter-optimized MPGA-ER to solve for more FJSP instances and demonstrate its effectiveness by comparing it with that of other algorithms proposed in other studies.” for more clarity. (see the words in red color in Abstract and Section 3.5 of the version named “Revised Manuscript with Track Changes”)

2. No consistency in writing for example in line 249 “(i) Machine crossover: two integers less than” and in line 251 “(ii) Operation crossover is POX crossover”. The same also in line 264 ‘(i) Machine mutation:” and no number in line 268 “Operation mutation:”

**Responses**: According to your comments, these descriptions have been revised for consistency. (see the words in red color in Sections 2.3.3 and 2.3.4 of the version named “Revised Manuscript with Track Changes”)

3. No consistency in writing for example in line 313 “Finally, the measures: average shortest path (ASP) and size of the maximum connected sub-graph (SMCS) are used to measure the ER network structures.”. However, the authors started describing SMCS first.

**Responses**: According to your comments, these descriptions have been revised for consistency. (see the words in red color in Section 2.4 of the version named “Revised Manuscript with Track Changes”)

4. In section 2.3, point (3) mutation: different crossover operators were presented and the caption of Figure 2 is Fig 2. Schematic diagram of a crossover. Which one of them is that?

**Responses**: The crossover operator you mentioned is the operation crossover. And the caption is changed to “Fig. 4. Schematic diagram of operation crossover.” in the revised manuscript. (see Fig. 4)

5. In abstract:”we study how the sub-population size and number and the propagation rate of advantageous genes affect the performance (evaluated by the average optimal value and success rate based on TIN) of MPGA-ER. Is it appropriate to insert the sentence “(evaluated by the average …) in this way?

**Responses**: According to your comments, this sentence has been changed to “we study how the sub-population number and size and the propagation rate of advantageous genes affect the performance of MPGA-ER, wherein the performance is evaluated by the average optimal value and success rate based on TIN.”(see the words in red color in Abstract of the version named “Revised Manuscript with Track Changes”)

6. In line 144, there is no reference for the mathematical model.

**Responses**: According to your comments, we have added two references to the mathematical model. (see the words in red color in Section 2.1 of the version named “Revised Manuscript with Track Changes”)

7. Symbols of FJSP should be tabulated.

**Responses**: According to your comments, the symbols used in the FJSP model are summarized in Table 2. (see Table 2)

8. The authors did not show a Gantt chart of an arbitrary schedule to the example presented in Table 2.

**Responses**: According to your comments, we have given a Gantt chart of the schedule represented by **F** ([2 1 4 4 0 4; 1 1 4 5 4 9; 2 2 5 5 4 9; 1 2 1 1 9 10; 2 3 4 2 9 11]) which is described in Section 2.3.2 in the example in Table 3, shown in Fig. 2. (see Fig. 2)

9. What is Jt in Line 214?

**Responses**: *J_t_* is the total number of operations of all jobs. According to your comments, we have explained *J_t_* in Table 2. (see Table 2)

10. What is POX crossover stands for?

**Responses**: POX crossover stands for the operation crossover. For consistency, the word “POX” is deleted in the revised manuscript. And we called it operation crossover directly. (see the words in red color in Section 2.3.3 of the version named “Revised Manuscript with Track Changes”)

11. The two-point crossover was not explained and a figure is needed to present it.

**Responses**: According to your comments, we have used a figure (Fig. 3) to explain the two-point crossover. (see Fig.3)

12. There is a reference ([27]) in line 251 (“Operation crossover is POX crossover [27]”). This refers to what.

**Responses**: This reference means that the POX crossover was described in [27]. As described above, the word “POX” is deleted in the revised manuscript, and we called it operation crossover directly for consistency. Then, we explain how to use it, which is also described in [27].

13. Subsections numbers of 2.3 section.

**Responses**: According to your comments, we have numbered all the subsections. (see Sections 2.3.1~2.3.4)

14. In line 307 it was mentioned” SR, which is also used to evaluate the performance of MPGA-ER”. However, this is the same as that in line 302.

**Responses**: According to your comments, we have deleted the redundant description. (see Section 2.4)

15. In line 303: “For a given TIN, every algorithm runs several times (denoted by Ntol) independently”. However, this number was not stated.

**Responses**: *N_tol_* is set to be 10 in this study. According to your comments, this description has been added in the revised manuscript. (see the words in red color in Section 2.4 of the version named “Revised Manuscript with Track Changes”)

16. A short description of how the instances were generated is preferred.

**Responses**: According to your comments, we have tried to explain why we choose these instances. (see the words in red color in Sections 3.1 and 3.5 of the version named “Revised Manuscript with Track Changes”)

Finally, thank you very much, and your comments are constructive and helpful.

Thank you and beat regards.

Yours sincerely

Xiaoqiu Shi; Wei Long; Yanyan Li; Dingshan Deng
